# Two Approaches to Increase Physical Activity for Preschool Children in Child Care Centers: A Matched-Pair Cluster-Randomized Trial

**DOI:** 10.3390/ijerph16204020

**Published:** 2019-10-21

**Authors:** Pooja S. Tandon, Katherine L. Downing, Brian E. Saelens, Dimitri A. Christakis

**Affiliations:** 1 Center for Child Health, Behavior, and Development, Seattle Children’s Research Institute, Seattle, WA 98145, USA; Brian.saelens@seattlechildrens.org (B.E.S.); Dimitri.christakis@seattlechildrens.org (D.A.C.); 2 Department of Pediatrics, University of Washington, Seattle, WA 98195, USA; 3 Institute for Physical Activity and Nutrition (IPAN), School of Exercise and Nutrition Sciences, Deakin University, Geelong VIC 3220, Australia; k.downing@deakin.edu.au

**Keywords:** physical activity, preschool, child care, outdoors, active play

## Abstract

Early childhood education settings are critical for promoting physical activity (PA) but intervention effects are often small. The aim of this study was to develop, test, and compare two approaches to increasing physical activity among preschoolers at child care centers: one focused on a teacher-led PA curriculum (Active Play!) and the other on increasing outdoor child-initiated free play time (Outdoor Play!). We conducted a matched-pair cluster-randomized study in 10 centers in and around Seattle, WA, USA (n = 97 children, mean age 4.6). Pre- and post-intervention data were collected from observations and accelerometers. At pre-intervention, 19% of Active Play! and 25% of Outdoor Play! children achieved >120 min/day of PA during child care. The total opportunity for PA increased in both interventions (Active Play! = 11 min/day; Outdoor Play! = 14 min/day), with the largest increase in outdoor child-initiated free playtime (Active Play! = 19 min/day; Outdoor Play! = 24 min/day). No changes in sedentary time, light or moderate- to vigorous-intensity PA (MVPA) were observed in either intervention and there was no difference between interventions in the percentage of children attaining more than 120 min/day of PA. A small (<3 min/day) relative increase in teacher-led outdoor activity was observed in the Active Play! intervention. Both intervention strategies led to an increase in active play opportunities, predominantly outdoors, but neither was able to substantially increase the intensity and/or duration of children’s PA. Future studies are needed to better understand and inform sustainable approaches to increase PA in early learning settings.

## 1. Introduction

Engaging in sufficient physical activity (PA) and limiting sedentary behavior (SB) are important for the health and development of preschool children (3 to 5 years), including benefits in lower adiposity, cardiometabolic health, motor skill development, psychosocial health, and cognition [1,2,3,4]. However, only approximately 50% of preschool children are meeting the US National Association for Sport and Physical Education (NASPE) [5] guidelines of at least 1 h of structured (teacher-led) and at least 1 h of unstructured (child-initiated) PA every day [6,7]. Given that the majority of preschool children in the US attend center-based child care [8], these settings offer critical intervention opportunities. Characteristics of child care centers, such as play equipment, outdoor time, and teacher practices have been associated with child PA [9,10,11,12,13,14]. 

Existing interventions in preschools and child care centers have predominantly used structured activity lessons implemented by external agents (e.g., researchers) to increase PA [15]. Although these interventions have been effective, the effects are often small and sustainability is limited [15]. Thus, it is prudent to investigate other more scalable strategies to increase PA. The best practice guidelines encourage a combination of teacher-led structured PA time and unstructured free play time, although how much each contributes to desirable active behavior is not known. Furthermore, different programs may be better suited or more amenable to one or the other approach. In fact, it may be ideal to do a combination of both approaches. However, since prior studies on the proposed interventions were lacking, this study focused on examining the efficacy of the two interventions separately.

The effects of increasing outdoor time as a strategy to increase PA in child care has not been extensively explored, despite time outdoors being shown to be positively associated with PA [16]. Outdoor time is also positively associated with children’s Vitamin D levels [17], motor development [18], vision [19], cognition [20,21], and mental health [22,23]. Yet, the amount of outdoor time may be suboptimal in child care [24], so increasing it may lead to more PA and numerous other benefits. A pilot intervention with low-income Latino children found that simply increasing preschool children’s outdoor free play time (by 1 h per day), without any additional intervention to change children’s activity, did not increase physical activity [25]. Therefore, research examining interventions that both increase outdoor time and help guide children to be more active while outdoors is warranted.

Another approach to increasing preschoolers’ PA may be to have teachers or other providers already in the child care setting provide more structured or guided physical activities for children in their care. A recent systematic review found that provider training was positively associated with a change in moderate- to vigorous-intensity PA (MVPA) in children aged 0–6 years [26]. However, evidence suggests that teacher-led active play is minimal in child care centers [24]. Adult-led physical activities have the added benefit of likely being more inclusive of all children and providing a setting for curricular instruction using movement. Training child care teachers in active play may be a more sustainable model to increase children’s PA than relying on outside trainers or experts.

The aim of this study was to test and compare two approaches to increasing total PA and decreasing sedentary time at child care centers, one which focused on increasing outdoor free play time (Outdoor Play!) and the other on a teacher-led PA curriculum (Active Play!). 

## 2. Materials and Methods 

### 2.1. Study Design

This was a matched-pair cluster-randomized study collecting data pre- and post-intervention (Clinicaltrials.gov Registration # NCT03752008). This study was approved by the Seattle Children’s Hospital Institutional Review Board. The full protocol is available upon request from the authors. 

### 2.2. Recruitment and Participants

Ten child care centers were recruited in the Seattle, WA area and were matched based on the demographics of the area where they were located. Matched pairs were then randomized (by flipping a coin) to receive either the Active Play! or Outdoor Play! intervention. Centers were eligible if they provided full (>6 h) day child care to preschool children (ages 3–5). The centers were part of a chain and therefore shared many similarities in terms of daily schedules, but all had their own leadership team and staff. All centers had adjacent outdoor play areas. The classroom with the oldest preschool-age children was chosen if the center had more than one classroom for 3–5 year olds.

Interventions and data collection occurred within the same time frames in matched centers. The study period went from October 2012 to April 2014. Baseline data were collected in the month prior to the workshop. Post-intervention data were collected at 12 weeks following the workshop. 

### 2.3. Interventions

Both interventions were similar in that they were multilevel and focused on improvements in the environment, the social context, and social support, although the content of each was different. In both interventions, teachers attended a 3-hour workshop and received supplementary materials relevant to the training. 

#### 2.3.1. Active Play! Intervention

This intervention focused on promoting PA in preschoolers through structured, teacher-led activities that could be easily incorporated into the daily curriculum without a specific focus on indoor or outdoor location. Teachers at participating centers attended one 3-hour workshop (delivered at the childcare center) that included a presentation and discussion of (1) the importance of daily physical activity for children’s health and development, (2) teachers’ beliefs and barriers to promoting PA, and (3) the Active Play! Intervention materials. Teachers were introduced to the Active Play! Fun Physical Activities for Young Children Book and DVD, which promotes PA and fundamental movement skills in young children [27], and were provided with two copies per center. Clips of the DVD demonstrating various activities were shown and activities were modeled by the trainer. Examples of activities included jumping on bubble wrap, hula hoop limbo, and an indoor obstacle course. The end of the book had a suggested curriculum that teachers could follow. Centers in the Active Play! intervention also received a set of portable toys needed for the suggested activities.

#### 2.3.2. Outdoor Play! Intervention

This intervention focused on promoting outdoor time in preschoolers as a means to increasing their PA, using both child-initiated and teacher-led activities. The training and materials were designed to emphasize the teacher’s own connection with nature, the benefits of outdoor time for children and adults, and problem solving around barriers encountered. Teachers at participating centers attended one 3-hour workshop (delivered at the childcare center) that focused on having participants recognize their own connection with the natural world and how to transmit that to the next generation. Teachers were given hats and gloves for themselves, a set of rain jackets and boots for the preschoolers, and ideas on what children could do outdoors to be active.

### 2.4. Measures

#### 2.4.1. Active Play Opportunities

A minimum of 4 full days of observation were undertaken per center, pre- and post-intervention by a trained researcher who was present between 08:00 and 17:00. The observation days occurred on the same days that children wore the accelerometers and observers went to the matched sites on the same days to account for weather conditions.

During direct observation, the observer noted the type of opportunity offered to children and noted whether it was an active play opportunity or not. Time periods were classed into one of six categories (see Table 1). More details about this methodology have been previously published [24].

#### 2.4.2. PA Levels

On observation days pre- and post-intervention, participating children were fitted with ActiGraph GT3X+ accelerometers (Actigraph, Pensacola, FL) on their right hip that they wore for the duration of the preschool day. The ActiGraph had been validated and calibrated for use among preschool children [28]. Data were collected in 15-second epochs, and sedentary time, light-intensity PA (LPA), and MVPA were classified using validated cut points [28]. Children were classified as meeting the guideline of 120 min/day of non-sedentary time during child care if they accumulated 120 min of MVPA per day. Physical activity data from the accelerometer were time-matched to observation data (categorized as described in Table 1). Although the observed type of activity reflects the opportunity offered to the preschoolers at that time, the activity level is quantified based on the children’s actual movements measured via accelerometer. Although the observation times and accelerometer wear time were similar, they were not necessarily equal (e.g., if a child took the accelerometer off for naptime or arrived later to child care). Hence, it is possible to have some sedentary time during ‘active opportunities’, or to have some MVPA during time classified as non-active or naptime.

#### 2.4.3. Demographics

Parents reported their child’s gender, date of birth, race/ethnicity, the highest educational attainment of the adults in the household, and the household income. Teachers were also asked demographic questions.

#### 2.4.4. Acceptability/Satisfaction

Directors/assistant directors and teachers attending the workshops (for both interventions) were asked to complete a survey at the end of the training workshop. 

### 2.5. Analyses

Analyses were conducted in Stata 14.0. All children with valid accelerometer data were included in the analyses. Continuous measures were summarized using means and SDs, and categorical variables were summarized using frequencies and proportions. Mixed-effects linear regression models were used to determine changes (post vs. pre-intervention) in outcomes. The primary outcomes considered were: total amount of time (min/day) and percent time spent in sedentary, LPA and MVPA while at childcare (from accelerometers), and total amount of time (min/day) and percent time in each of the six activity categories (from observation). Additionally, from the direct observation data, total time spent outdoors (sum of teacher-led and child-initiated outdoor activity), total time indoors (sum of teacher-led and child-initiated indoor activity, non-active time and naptime), and total active opportunity time (sum of teacher-led and child-initiated outdoor and indoor activity) were calculated. Non-active time was included in total time indoors as we did not encounter any outdoor time that would fit in the non-active opportunity category. Mixed-effects linear regression models were also used to compare the effects of two interventions, including a time-by-condition interaction effect. We used separate mixed-effects linear regression models to test the effect of time in the six categories on percent time in sedentary, LPA and MVPA. All analyses were controlled for child gender and age, and were adjusted for clustering of children within child care centers. As appropriate, analyses also controlled for accelerometer wear time or total observation time.

## 3. Results

Eight to fourteen children from one preschool classroom in each of the 10 centers were recruited to participate in the study. Demographic data were collected from 82 children (35 Active Play!, 47 Outdoor Play!) from 10 centers (5 Active Play!, 5 Outdoor Play!) and 14 teachers; characteristics are presented in Table 2. Not all participants provided demographic information.

### 3.1. Child Physical Activity

Table 3 and Table 4 present children’s mean minutes per day in PA (overall and separated by opportunities, respectively) in the Active Play! and Outdoor Play! centers pre- and post-intervention.

#### 3.1.1. Pre-Intervention

(1) PA levels

Children in both interventions spent approximately 280 min/day in sedentary time, 50 min/day in LPA, and 50 min/day in MVPA pre-intervention (Table 3). Nineteen percent of Active Play! and 25% of Outdoor Play! children met the guideline of 120 min of non-sedentary time (i.e., LPA and MVPA) per day using accelerometry data. 

(2) Active play opportunities

Pre-intervention, children in both interventions spent approximately 88% of the day in activities not providing opportunity for PA (Table 4). There were differences at pre-intervention between the two intervention groups in non-active opportunities (*p* < 0.001), outdoor child-initiated active play opportunities (*p* = 0.005), total outdoor time (*p* = 0.006), and naptime (*p* < 0.001).

#### 3.1.2. Post-Intervention

(1) PA levels

No pre- to post-intervention changes in children’s sedentary time, LPA or MVPA were observed in either the Active Play! or Outdoor Play! Interventions and the interaction of time by condition was not significant (Table 3). Additionally, no significant pre to post changes were observed in the percentage of children meeting the 120 min/day PA guideline at post-intervention. 

(2) Active play opportunities

In the centers receiving the Active Play! intervention, total opportunity for PA increased by an average of 11 min/day (2.5% of the day; *p* = 0.002); see Table 4. Outdoor child-initiated and teacher-led activity both increased by 19 min/day (*p* < 0.001) and 2 min/day (*p* = 0.04), respectively. Conversely, indoor child-initiated activity decreased by 8 min/day (*p* < 0.001). Total outdoor time increased by 21 min/day, while total indoor time decreased by 19 min/day (both *p* < 0.001). No significant changes from pre-intervention to post-intervention were observed in min/day of any of the other observation categories.

For children in centers receiving the Outdoor Play! intervention, total opportunity for PA increased by an average of 14 min/day (3.0%; *p* < 0.001) and naptime increased by 8 min/day (*p* = 0.03). Total non-active time decreased by 22 min/day (*p* < 0.001), while total indoor time significantly decreased by 24 min/day, seemingly replaced by total outdoor time which significantly increased by 24 min/day (both *p* < 0.001). Indoor child-initiated activity decreased by 5.5 min/day (*p* < 0.001).

The only significant differences observed post-intervention between the interventions favored Active Play! and were for outdoor teacher-led time (3 min difference; *p* = 0.008), and indoor teacher-led activity (4 min difference; *p* = 0.03).

#### 3.1.3. Characterization of Children’s PA Levels by PA Opportunities

When we examined the characterization of children’s time (percent time sedentary, in LPA and in MVPA) in each of the PA opportunity observation categories, and for total outdoor and indoor time, results showed that from pre- to post-intervention in the Active Play! intervention, children’s LPA increased by 6.5% in indoor child-initiated activity (*p* = 0.02). For children in the Outdoor Play! intervention, percent time sedentary for total indoor time increased by 3.5% (*p* = 0.01), while percent time in LPA decreased by 1.5% (*p* = 0.006), and percent time in MVPA decreased by 1.9% (*p* = 0.03) during indoor time. Additionally, percent time in LPA decreased in outdoor teacher-led activity by 12.2% (*p* = 0.02) and in total non-active time by 1.5% (*p* = 0.01). No other changes were observed in percent time in sedentary, LPA and MVPA in any of the other categories for either intervention group. 

### 3.2. Acceptability/Satisfaction

A total of 23 preschool staff attending the Active Play! workshops and 17 attending the Outdoor Play! workshops completed the feasibility/satisfaction questionnaire. The vast majority (96%) of those attending the Active Play! workshops reported that they were ‘very’ useful, while 71% of those attending the Outdoor Play! workshops reported that they were ‘very’ useful. All Active Play! workshop attendees and 94% of Outdoor Play! workshop attendees reported that they planned to practice what they had learned. 

## 4. Discussion

This study found that both intervention strategies led to an increase in active play opportunities, predominantly outdoors, but no changes in the children’s objectively measured LPA or MVPA were observed in either group. Interestingly, the outdoor child-initiated play time increased almost identically in both interventions, even though it was the specific focus of only the Outdoor Play! intervention. A small (<3 min/day) relative increase in teacher-led outdoor activity was observed in the Active Play! intervention, which targeted teacher-led activity. The overall increase in active play opportunities is encouraging and it could be that intervention participation (regardless of intervention condition) prompted teachers to increase what is most familiar to them in terms of providing more active play opportunities. Interestingly, in our study we found that despite total outdoor time increasing by about 20 min in both groups, there were no increases in percent of time in LPA and MVPA during this time; in fact, LPA decreased by about 12% during outdoor teacher-led activity in the Outdoor Play! group. Potentially, the focus for future interventions needs to be training teachers to provide higher intensity physical activity opportunities, rather than just active play and outdoor play opportunities.

Outdoor child-initiated play (i.e., ‘recess’-like) is typically the most common active play opportunity offered to children in early childhood education settings [24]. Research suggests that there are numerous benefits for children from outdoor time [17,18,19,20,21,22,23] and from child-initiated play [29], so an increase in these is likely valuable; however, in the present study it was not sufficient to increase the MVPA of preschoolers. We also saw a decrease in child-initiated indoor time in both intervention groups, which could have been a seasonal effect and mitigated the increase in total and outdoor active play opportunities.

Post-intervention, children in both groups were receiving less than an hour of outdoor play time daily, which is considerably less than the recommended 60–90 min/day [16]. While outdoor time has been found to be associated with PA, few interventions have specifically focused on increasing outdoor time. Our findings are consistent with those of the study by Alhassan et al. [25], which found that increasing preschoolers’ outdoor recess time from 60 to 120 min for two days did not increase their accelerometer-measured PA. Since outdoor time has numerous other benefits for children’s health and development, efforts to increase children’s exposure to the outdoors (and specifically nature-rich environments) are warranted. However, additional research is needed on how to best structure this time for it to be conducive to promoting more PA. For example, we did not prescribe how the increase in outdoor time be scheduled; perhaps smaller but more frequent opportunities to be outside would be more effective since children are more active in the first 10–15 min of outdoor time [30,31]. Also, having a more robust teacher-led component could be valuable while maintaining some child-initiated play time. In the Active Play! Group, although outdoor teacher-led time increased, it was likely too minimal to conclude whether increased adult-led, structured outdoor time would lead to increased intensity and/or duration of PA in preschoolers.

Our intervention provided a single session of this training for the providers and provided some relevant equipment to try to address commonly reported barriers to increasing physical activity. In Outdoor Play!, we provided rain coats, boots, and warm clothing; in Active Play!, we provided numerous items that could be used for indoor active play per the curriculum provided. It is difficult to ascertain from our study design if the provision of training vs. equipment/gear was the reason for the increase in active play opportunities, or if both were needed. Regardless, despite being well-received, neither intervention was able to substantially modify teacher-led physical activities or the intensity and/or duration of PA in preschoolers.

It is likely that the low intensity of the interventions contributed to a lack of change in children’s PA. Previous intervention strategies in preschools, even those with greater intensity, have also been largely unsuccessful in achieving substantial and sustained increases in young children’s PA [32]. A review of existing PA interventions in child care found the most consistent improvements by implementing structured PA programs [33]. A more recent intervention (SHAPES) based on a flexible ecological model focused on educator training and coaching—was effective in modestly increasing preschoolers’ MVPA during the preschool day by 0.8 min/h but this did not translate into a significant increase in total PA (there was a decrease in light activity) [34]. The model of providing teacher training, while a common method of imparting information to educators and typically required for their professional development, may not be effective in an environment where educators are busy, have numerous competing priorities (including academics’ and children’s safety), and often a high staff turnover. In the model where outside experts come in to regularly lead physical activities, limited resources often prevent sustainability. Perhaps interventions that integrate both teacher training with expert coaching/support would provide a hybrid model that is both effective and sustainable. One model for this in the U.S. would be to incorporate PA metrics into widely-adopted, state-wide quality rating and improvement systems (QRIS) that typically focus on the quality of early childhood education with the support of coaches/consultants but have not systematically included health behaviors [35].

This study has some limitations that need to be considered, particularly with regard to the generalizability of the findings. The study sample was relatively small, recruited from one geographic area, and there were some significant differences between the groups at pre-intervention despite matching centers based on area demographics. In addition, the increase in outdoor time in both cohorts could represent a seasonal effect. However, the two interventions were delivered in temporally matched seasons and over two years, in order to line up with the academic calendar, the pre-study assessments occurred in the fall, with the intervention training and delivery of materials occurring in the winter, and the post-assessments occurring in the spring. Strengths include a rigorous study design including concurrent direct observation and accelerometry. Future studies with larger samples, longer follow-ups, and more intense interventions and measurements of implementations are needed to better understand and inform sustainable approaches to increase PA in early learning settings.

## 5. Conclusions

Both intervention strategies (one focused on promoting outdoor play, the other on teacher-led play), led to an increase in active play opportunities, predominantly outdoors, but neither was able to substantially increase the intensity and/or duration of children’s PA. Future studies with larger samples, longer follow-ups, and more intense interventions and measurements of implementations are needed to better understand and inform sustainable approaches to increase PA in early learning settings.

## Figures and Tables

**Table 1:** Categories of observed activities.

Category	Description	Examples
**No Opportunities for PA**		
1. Non-active time	All indoor/outdoor structured and non-structured ‘non-active’ activities	Circle time, seated learning activities, meals
2. Naptime	Children required to sleep or lay on a mat for ‘quiet’ activities	Naptime
**Opportunities for PA**		
3. Outdoor child-initiated activity	Children free to choose activities outdoors	Free play in playground
4. Outdoor teacher-led activity	Teacher-led activities outdoors	Running laps, active games; all children expected to participate
5. Indoor child-initiated activity	Active play encouraged indoors with children initiating activities	Climbing equipment, balls (in an indoor playroom)
6. Indoor teacher-led activity	Teacher-led activities indoors	Yoga, ball games; all children expected to participate

**Table 2:** Child and teacher demographic characteristics, % unless otherwise noted.

	Active Play!	Outdoor Play!	*p*-Value *
Child	(n = 35)	(n = 47)	
Female	61.3	52.5	0.32
Age, mean (SD)	4.5 (0.6)	4.6 (0.4)	
Race/ethnicity			
White	57.1	63.8	0.54
African American/ black	5.7	4.3	0.76
Asian/ Native American/ Pacific Islander	8.6	4.3	0.42
Hispanic	5.7	2.1	0.39
>1 race	22.9	25.5	0.78
Highest educational attainment in household			
Less than high school	0	4.3	0.22
Completed high school	24.2	25.5	0.78
Completed college	51.5	53.2	0.68
Graduate/professional degree	24.2	17.0	0.51
Household income, $			
≤29,000	27.3	34.1	0.54
30,000-49,000	27.3	9.1	0.04
50,000-69,000	0.0	9.1	0.08
70,000-89,000	15.0	11.4	0.82
≥90,000	30.3	36.4	0.60
Teacher	(n = 6)	(n = 8)	
Female	100	100	-
Age, mean (SD)	32.0 (14.1)	39.8 (12.5)	0.32
Race			
White	16.7	75.0	0.03
African American/ black	16.7	12.5	0.83
Asian/ Native American/ Pacific Islander	33.4	12.5	0.35
Hispanic	33.3	0.0	0.08
Highest educational attainment			
Completed high school	66.7	87.5	0.35
Completed college	0.0	12.5	0.37
Graduate/professional degree	33.3	0.0	0.08

* differences between groups.

**Table 3:** Comparison of accelerometer-measured ^1^ sedentary time and physical activity by intervention type.

Min/day	Active Play! (n = 43)			Outdoor Play! (n = 54)			Adjusted Diff between Groups ^2^
	Pre Mean (SD)	Post Mean (SD)	Adjusted Change Mean (95% CI) ^2^	Pre Mean (SD)	Post Mean (SD)	Adjusted Change Mean (95% CI) ^2^	
Sedentary time	276.5 (41.9)	256.6 (50.9)	−8.4 (−19.2, 2.3)	281.2 (50.2)	268.2 (54.7)	2.6 (−7.6, 12.9)	3.4 (−11.3, 18.1)
LPA	46.6 (12.4) *	44.7 (11.3)	2.5 (−1.8, 6.7)	51.5 (11.6) *	47.0 (13.1)	−3.1 (−7.1, 0.9)	−3.0 (−8.7, 2.7)
MVPA	51.1 (18.0)	53.2 (16.4)	5.7 (−2.1, 13.4)	56.7 (17.9)	57.8 (20.1)	0.4 (−6.6, 7.5)	−0.5 (−10.8, 9.8)

Notes: * Denotes statistically significant difference between groups pre-intervention; ^1^ Average accelerometer wear time was 374.2 and 391.4 min per day pre-intervention for the Active Play! and Outdoor Play! interventions, respectively, and 354.5 and 373.0 min per day post-intervention for the Active Play! and Outdoor Play! interventions, respectively; ^2^ Adjusted for gender, age, accelerometer wear time, and clustering by child care center.

**Table 4:** Comparison of direct observation ^1^ variables by intervention type.

Min/day	Active Play! (n = 43)			Outdoor Play! (n = 54)			Adjusted Diff between Groups ^2^
	Pre Mean (SD)	Post Mean (SD)	Adjusted Change Mean (95% CI) ^2^	Pre Mean (SD)	Post Mean (SD)	Adjusted Change Mean (95% CI) ^2^	
No opportunity for PA, total	382.1 (52.6)	366.7 (62.3)	**−8.4 (−16.4, −0.5)**	380.0 (54.5)	361.3 (57.1)	**−13.4 (−17.6, −9.3)**	0.8 (−7.2, 8.7)
Non-active time	251.5 (41.7) *	231.4 (48.8)	−12.4 (−25.8, 0.9)	282.0 (42.8) *	257.3 (49.4)	**−21.6 (−29.0, −14.1)**	0.3 (−13.4, 14.1)
Naptime	130.6 (30.5) *	135.3 (31.0)	3.2 (−6.9, 13.3)	98.0 (25.8) *	104.0 (27.5)	**8.0 (0.8, 15.2)**	0.5 (−11.0, 11.9)
Opportunity for PA, total	46.1 (15.1)	61.8 (27.4)	**11.3 (4.0, 18.7)**	50.4 (13.7)	59.7 (13.8)	**13.7 (9.7, 17.8)**	−2.8 (−10.4, 4.8)
Outdoor child-initiated	27.8 (10.9) *	48.6 (24.2)	**18.8 (12.6, 25.0)**	35.8 (14.6) *	55.9 (14.4)	**23.8 (19.1, 28.4)**	2.0 (−5.1, 9.1)
Outdoor teacher-led	0.4 (1.2)	3.1 (7.4)	**2.5 (0.1, 4.9)**	0.4 (0.9)	0.4 (0.8)	0.1 (−0.2, 0.5)	**−2.6 (−4.5, −0.7)**
Indoor child-initiated	9.0 (9.4)	2.4 (3.8)	**−8.2 (−11.5, −4.9)**	6.1 (7.2)	0.0 (0.0)	**−5.5 (−7.3, −3.7)**	1.5 (−1.8, 4.8)
Indoor teacher-led	8.8 (11.0)	7.7 (6.3)	−1.9 (−5.1, 1.4)	8.2 (6.7)	3.4 (4.2)	**−4.7 (−6.5, −2.9)**	**−3.7 (−6.9, −0.4)**
Total outdoor time ^3^	28.3 (10.7) *	51.7 (26)	**21.4 (14.6, 28.3)**	36.1 (15.0) *	56.3 (14.4)	**23.9 (19.3, 28.6)**	−0.6 (−8.1, 6.9)
Total indoor time ^4^	399.9 (56.3)	376.7 (63.9)	**−18.7 (−27.0, −10.5)**	394.3 (55.8)	364.7 (58.2)	**−23.6 (−28.4, −18.9)**	−1.3 (−9.7, 7.1)

Notes: Bold font denotes statistical significance in change pre to post; * Denotes statistically significant difference between groups pre-intervention; ^1^ Average direct observation time was 432.5 and 430.2 min per day pre-intervention for the Active Play! and Outdoor Play! interventions, respectively, and 430.0 and 421.0 min per day post-intervention for the Active Play! and Outdoor Play! interventions, respectively; ^2^ Adjusted for gender, age, total observation time, and clustering by child care center; ^3^ Sum of outdoor child-initiated and teacher-led active time; ^4^ Sum of indoor child-initiated and teacher-led active time, non-active time and naptime.
